# 

**Published:** 1872-01

**Authors:** 


					﻿PUBLISHED MONTHLY, AT $3 PER ANNUM, IN ADVANCE,
i
,	ATLAXT2V
S
•2	§'■
-e
I	i>'
i Medical and SurgicalJoumLl
§1	•	>3
i	l-g
•s	s-
g i	___________ ' *
d I	.®
2
«	'I
'5 ■
EDITED BY
P<	g;
I	J. P. LOGAK, M.D.,
5	I-?
rt	AND	. a-
S
AV. F. WFSTAIORELAND, M.D.,	|
i	5^
S : Professor of the Principles and Practice of Sxiryery in Atlanta Medical College. ; g
o ■	•	‘ p
g	.
>1 ------------------------- .
<P<xx et Scientia, sed, veritas sin,e timore.
S;	--------------- !»
® :	r.-
d	®
rt'	■§
I i	VOLUAIE IXB-JANUARY, 1872.-NUMBER 10.	! |
<a*i	«
;21
■a	I
o	®
§	—:
-§	•
eo
I	ATLANTA, GEORGIA:	|
H	5“
S PLANTATION PUBLISHING COAIPANY.
Ph
'	1871.
EACH NUMBER CONTAINS SIXTY-FOUR PACES.
				

